# Improving tuberculosis diagnosis: Better tests or better healthcare?

**DOI:** 10.1371/journal.pmed.1002406

**Published:** 2017-10-17

**Authors:** Sumona Datta, Matthew J. Saunders, Marco A. Tovar, Carlton A. Evans

**Affiliations:** 1 Infectious Diseases & Immunity, Imperial College London, and Wellcome Trust Imperial College Centre for Global Health Research, London, United Kingdom; 2 Innovación Por la Salud Y Desarrollo (IPSYD), Asociación Benéfica PRISMA, Lima, Perú; 3 Innovation For Health And Development (IFHAD), Laboratory of Research and Development, Universidad Peruana Cayetano Heredia, Lima, Perú

## Abstract

In a Perspective accompanying Sylvia and colleagues, Carlton Evans and colleagues discuss the challenge of squaring policies around tuberculosis diagnosis with the realities of clinical practice in small villages and low-resource settings.

Tuberculosis (TB) is a preventable and curable disease, but it kills more people than any other infection. Many people with TB are never diagnosed, and those who are diagnosed are often ill and contagious for many weeks or months before a diagnosis is made. Barriers to TB diagnosis are well described, often including poverty; stigma; marginalization; indolent, nonspecific symptoms; and poorly performing diagnostic tests. However, despite their central role in TB diagnosis, healthcare providers have been the subject of surprisingly little research [1].

This week in *PLOS Medicine*, Sylvia and colleagues report findings with important implications for TB elimination [1]. They trained and sent simulated “standardized patients,” also known as “mystery clients,” to healthcare providers at village clinics, township health centers, and county hospitals in China and found that the care provided in 274 consultations differed greatly from TB recommendations. The standardized patients reported classical TB symptoms, but only 15% of the providers mentioned TB, and only 41% of the providers tested or referred patients as recommended for TB. These differences between policy and practice were especially marked in the village clinics where most care was provided, and simulations suggested that a proposed system of managed referral with gatekeeping at the level of the village clinic would further reduce correct management, all of which makes for uncomfortable reading.

## The “know-do gap”

Perhaps the most remarkable aspect of this study is that although the providers did not generally manage patients with typical TB symptoms as recommended, when village and township doctors were presented with the same symptoms described in clinical vignettes, 81% were managed according to TB recommendations. Thus, as in the authors’ previous research [2], the providers generally seemed to know what policies recommended but in practice usually did something quite different. This “know-do gap” is a common observation in quality improvement studies, and research to understand the reasons for it is a priority for TB elimination. Closing the TB know-do gap will surely be more complex than knowledge transfer and should consider systems constraints, environmental factors, and personal experiences that impact human behavior [3].

## Tuberculosis testing policy-practice gap

TB policies generally recommend sputum testing for diagnosing pulmonary TB [4], whereas in this study, X-rays were more popular. This policy-practice gap is more complex than a shortfall in practice, partly because sputum TB testing is more likely to be stigmatized and is less sensitive than X-rays. Providers said that they would do the recommended sputum testing, which is specific but insensitive, whereas in practice they did more sensitive but less specific X-rays. Amongst the potential explanations, the authors discuss the possibility of conflicts of interest encouraging tests and treatments that benefit providers financially. If such conflicts of interest exist, then they should urgently be disclosed and removed, but no evidence is presented that these are common. Perhaps more importantly, these providers were likely not focused on TB diagnostic guidelines (which prioritize sputum testing) but rather were trying to establish what was wrong with unwell people (which may be facilitated more by an X-ray than a sputum TB test). We need additional, specific research to better understand why providers said that they would do recommended TB tests but, despite their availability, chose to do other tests. Recommendations may need to be changed, sputum TB testing may need to be offered in a more accessible and acceptable way, and interventions may need to be developed to help evidence-based policies drive practice.

## Poor quality or real-world care?

Because it did not match recommendations, Sylvia and colleagues describe the care usually provided as “incorrect” and of “poor quality” with frequent “quality deficits.” But is this research really an exposé of providers not doing what they should or of suboptimal policies and of tests provided in a suboptimal way? Primary care practitioners may see several patients per day whose symptoms could be caused by TB but see a new patient with TB only every few months or years. As the authors state, in most real-world settings, even the classical TB symptoms reported by the standardized patients would in most cases be caused by a simple chest infection, not TB. Were the providers sometimes right to treat the standardized patients initially as a simple chest infection, assuming that patients would return if they had a less common, persistent illness like TB? Did providers consider TB a possible diagnosis (as the clinical vignette findings would seem to suggest) but not mention or act upon this unlikely possibility during first consultations to avoid causing patients costs and stigma? Future research should address a key limitation of this study by including follow-up consultations with standardized patients, which is important because providers may behave differently when patients return with persistent symptoms because their illness was not adequately addressed by their first consultation. Importantly, sputum TB testing should be available in a local, decentralized setting, without causing patient dissatisfaction due to direct or indirect costs [5], and should also be available in a discreet manner to avoid stigmatization, fear, and marginalization impairing patient’s social life and employment [6]. This may be achieved by enabling providers to send sputum directly for “acid fast microscopy” rather than referring people for reassessment by a distant TB program. These simple optimizations of how TB testing is offered should help providers to follow TB recommendations, facilitating early sputum TB testing of all patients with prolonged cough whilst also accommodating the needs and priorities of patients, the great majority of whom do not have TB.

## Tuberculosis programs facilitating integrated care

Sylvia and colleagues’ findings highlight the need to better integrate TB diagnosis with primary care algorithms and with the priorities of universal health coverage. TB is uncommon and has nonspecific symptoms, so TB case finding may be helped more by thoughtful inclusion of TB testing integrated within primary care pathways rather than by the current emphasis on separate programmatic TB case-finding policies. Furthermore, modern healthcare is afflicted by a plague of guidelines, policies, recommendations, international standards, and algorithms. These have poor concordance, variable quality, inadequate evidence-base, and inconsistent updating systems but are unified by complexity that impairs utility [7]. The generalists who must diagnose and care for the great majority of individuals with TB need simple, pragmatic, and syndromic policies to support the integrated care of people experiencing symptoms more than multiple disease-specific guidelines.

## The long journey to tuberculosis diagnosis

Few individuals working with people living with TB will be surprised by these findings, since many patients with TB experience repeated missed opportunities for TB diagnosis, often causing worsening illness and contagion to others [8]. Furthermore, prevalence surveys have demonstrated that many people who have TB disease have not engaged with healthcare providers at all [9]. Thus, community engagement will be important for increasing awareness and provider accountability as well as for health system strengthening and addressing the social determinants of TB and of access to TB care [10].

## Tuberculosis policy for the real world

Perhaps the greatest value of this research transcends the above points. A survey of TB research publications or the agenda for TB conferences give the impression that TB elimination depends upon the development of better tests and pills, whilst TB policy documents understandably describe a perfect model of optimal care. Sylvia and colleagues [1] are to be commended for highlighting and characterizing the profound discord between these currently unrealizable goals, contrasted with the real-world experience of people with typical TB symptoms who are unlikely to receive early TB testing. TB principally affects poorer people who are cared for in poorly resourced places. Thus, to be effective, better tests, pills, and TB policies should be integrated with interventions addressing the factors limiting access to TB care and urgently require a greater emphasis on assessing and improving TB care as an integrated component of the basic healthcare that people receive in the real world.

## Abbreviation

TB: tuberculosis

## References

[1] Sylvia S, Xue H, Zhou C, Shi Y, Yi H, Zhou H, et al Tuberculosis detection and the challenges of integrated care in rural China: A cross-sectional standardized patient study. PLoS Med. 2017;14(10): e1002405. 10.1371/journal.pmed.1002405 PMC5644979 29040263

[2] Das J, Kwan A, Daniels B, Satyanarayana S, Subbaraman R, Bergkvist S, et al Use of standardised patients to assess quality of tuberculosis care: a pilot, cross-sectional study. Lancet Infect Dis. 2015;15: 1305–13 doi: 10.1016/S1473-3099(15)00077-8 26268690 10.1016/S1473-3099(15)00077-8 PMC4633317

[3] Bandura A. Social cognitive theory of personality In: Pervin L & John O, editors. Handbook of personality 2^nd^ ed New York: Guilford Publications; 1999 pp 154–196.

[4] Evans CA. GeneXpert—A Game-Changer for Tuberculosis Control? PLoS Med. 2011;8(7): e1001064 doi: 10.1371/journal.pmed.1001064 21814497 10.1371/journal.pmed.1001064 PMC3144196

[5] Wingfield T, Boccia D, Tovar MA, Gavino A, Zevallos K, Montoya R, et al Defining catastrophic costs and comparing their importance for adverse TB outcome with multi-drug resistance: a prospective cohort study, Peru. PLoS Med. 2014;11(7): e1001675 doi: 10.1371/journal.pmed.1001675 25025331 10.1371/journal.pmed.1001675 PMC4098993

[6] Rocha C, Montoya R, Zevallos K, Curatola A, Ynga W, Franco J, et al The Innovative socioeconomic interventions against TB (ISIAT) project–an operational assessment. Int J Tuberc Lung Dis. 2011;15(Suppl 2): S50–7 21740659 10.5588/ijtld.10.0447 PMC3160483

[7] Datta S, Shah L, Gilman RH, Evans CA. Comparison of sputum collection methods for tuberculosis diagnosis: a systematic review and pairwise and network meta-analysis. Lancet Glob Health. 2017;5: e760–771. doi: 10.1016/S2214-109X(17)30201-2 28625793 10.1016/S2214-109X(17)30201-2 PMC5567202

[8] Subbaraman R, Nathavitharana RR, Satyanarayana S, Pai M, Thomas BE, Chadha VK, et al The Tuberculosis Cascade of Care in India's Public Sector: A Systematic Review and Meta-analysis. PLoS Med. 2016;13(10): e1002149 doi: 10.1371/journal.pmed.1002149 27780217 10.1371/journal.pmed.1002149 PMC5079571

[9] WHO. Tuberculosis prevalence surveys: a handbook. World health Organization, Geneva; 2011.

[10] Wingfield T, Tovar MA, Huff D, Boccia D, Montoya R, Ramos E, et al A randomized controlled study of socioeconomic support to enhance tuberculosis prevention and treatment, Peru. Bull World Health Organ. 2017;95(4): 270–280. doi: 10.2471/BLT.16.170167 28479622 10.2471/BLT.16.170167 PMC5407248

